# Current trends in palm oil waste management: A comparative review of Cameroon and Malaysia

**DOI:** 10.1016/j.heliyon.2023.e21410

**Published:** 2023-10-30

**Authors:** Egbe Terence Awoh, Joseph Kiplagat, Stephen K. Kimutai, Achisa C. Mecha

**Affiliations:** a Department of Mechanical, Production and Energy Engineering, School of Engineering, Moi University, P.O. Box 3900, Eldoret 30100, Kenya; b Renewable Energy, Environment, Nanomaterials, And Water Research Group, Department of Chemical and Process Engineering, Moi University, P.O Box 3900, Eldoret 30100, Kenya; c Department of Electrical and Electronic Engineering, Faculty of Engineering and Technology, University of Buea, P.O Box 63, Buea, Cameroon

**Keywords:** Sustainability, Feasibility, Waste management, Palm biomass, Cameroon

## Abstract

This paper carried out a comparative review on the current trends in the conversion of palm oil waste into value-adding products by the Cameroonian and Malaysian palm sectors/researchers. Trends like composting, composite, pulping, mushroom cultivation, pyrolysis, aerobic and anaerobic digestion of palm biomass were studied as means to reduce the bulk, and to curb emissions of Greenhouse gas while producing value. Base on this research, limited works has been done on the conversion of palm biomass into value in Cameroon, whereas Malaysian palm researchers have employed all of these techniques and producing values from them. It was discovered that the various conversion process have different degree of feasibility and sustainability, and the end-products have different applications. Conversion process like pyrolysis is relatively faster, it could take just a few minute and the end-product which is biofuel have a wide range of applications; in contrast to composting which could take up to 180 days to mature and the end-product is limited to fertilizer. This research aims to sensitize the palm sector in Cameroon to the various processes that can be applied to sustainably manage palm waste. A priority table was also developed based on the feasibility and sustainability of the various conversion processes to serve as a guide towards sustainable waste management in the agro-industrial palm sector in Cameroon and a step towards industrialization.

## Highlights

• Management of palm waste by composting, pulping, composite, fungiculture and biofuel.
• Sustainability of these conversion processes.
• Feasibility of these conversion processes.
• The value adding from these conversion processes.

## Graphical abstract

**Figure:**
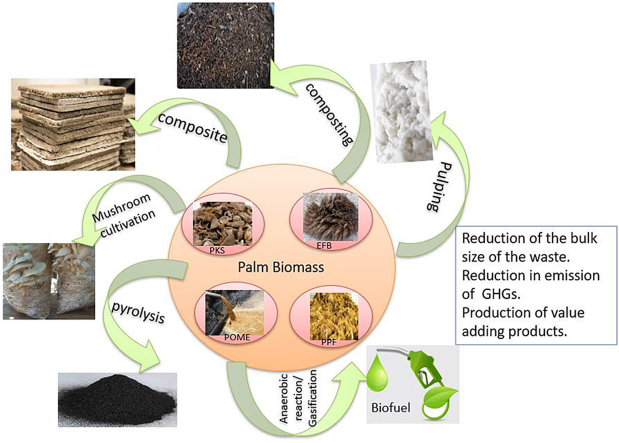

## 1 Introduction

Palm oil (*Elaeis guineensis*) originated in West Africa, around the great tropical forest bordering the Gulf of Guinea where it grew naturally and was also cultivated. The indigenous people adopted the name “god-given gift” because of the variety of uses it plays in their everyday life. It was and remains till today the main source of edible oil (palm oil and palm kernel oil) and wine (palm wine) for the indigenous people of West Africa. Apart from using it as a source of oil and wine, the leaves (are used for roofing and sweeping), trunk (for building and fencing), roots (for medicinal purpose), biomass (for cooking), ashes (fertilizers) and soap among others [1,2].

Today, there are so many palm agro-industrial companies in countries with equatorial climate. This is because palm oil have the highest yield per hectares of all edible oil, which is about four times rapeseed, producing a total of 39.6 % of the total vegetable oil (oilworld, 2019); it is also the cheapest and most consumed form of vegetable oil (statista, 2023). This makes palm oil the most popular form of vegetable oil in the market. However, the sustainability of palm oil production is hunted by two major issues; (i) expansion and (ii) waste management. Despite the productivity of palm oil, it can only give maximum yield when grown under equatorial climate and most of the equatorial rainforest had already been cleared for this purpose (nationalgeographic, 2018). Hence, further expansion will mean further destruction of the already endangered rainforest, and the diverse animals and plants life in it. That is why palm investors from these countries are moving their expansion to Cameroon, among other African countries [1,3].

In contrast, researches on palm waste management in Cameroon are quite lacking as compare to Malaysia, and expansion on the palm sector will spell more waste. There are four main types of palm waste from palm oil production process, which total 80 % of the process. Of the four, only palm pressed fiber (PPF) and palm kernel shell (PKS) are being used sustainably in Cameroon. These two are being used to heat-up the boilers and provide auxiliary energy in the mills. However, empty palm bunches (EFBs) and POMEs are being unsustainably disposed to the environment closed to the mills and not much is being said on the treatment. They both have adverse effect to the environment; EFBs decompose to emit greenhouse gases (GHGs) and pollute underground water, while POME being disposed at temperature higher than environmental temperature causes thermal shock and emissions. With more investors coming into palm sector, so too will the issue of palm waste management increases [3]. Palm oil giants like SOCAPALM (*Société Camerounaise de Palmeraies*) claims to be utilizing all of its waste, with the exemption of POMEs which are being stored in catchment ponds to be treated anaerobically and aerobically. Despite this, there is no methane capture facility to convert this gas to biogas, instead the gas is allowed into the atmosphere; couple with fact that many NGOs and locals are still accusing SOCAPALM of pollution (socapalm, 2020). This is the same complain about the other agro-industrial palm producers in Cameroon. In spite of the high potential derived from the utilization of palm waste, EFBs are only used for mulching; a process by its own is not sustainable. POMEs are allowed to contaminate near-by land and water bodies, creating bio-hazard and destroying the environment. So, there is a need to sensitize the palm sector and the local communities in Cameroon on the opportunities that can be seized from using palm biomass as precursor.

## 2 Biomass from palm oil production

After harvesting the fresh fruit bunches (FFB) from the palm trees, the palm fruits are boiled and trip-off the bunches leaving behind the empty palm bunches (EFB) before processing by mashing and pressing to extract the crude palm oil (CPO). Palm kernel oil is gotten after the palm nuts from the palm fruits are cracked and further processed by heating, and is richer in saturated fat as compared to CPO.

Waste from oil palm cultivation and palm oil processing include empty fruit bunches (EFB), palm kernel shell (PKS), palm pressed fiber (PPF), sludge and palm oil mill effluent (POME) [4]. It is estimated that one ton of FFB produces 21 % of CPO, 23 % EFB, 14–15 % PPF and about 6–7 % PKS [5,6].

**Fig. 1:**
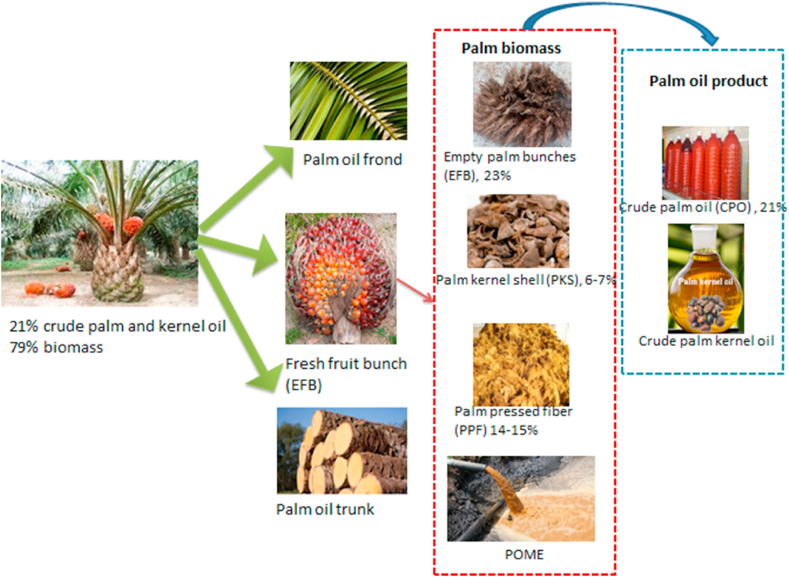
Palm oil product and biomass from the production process.

In the actual sense, only the EFB and POME are considered as waste by the mills since PPF and PKS are almost completely used-up in heating the boilers and providing auxiliary energy [7]. Fig. 1 below deputes the various wastes from the palm oil process including the crude palm and kernel oil.

Previous studies on the ultimate analysis of oil palm biomass showed the existence of Carbon (C), Hydrogen (H), Nitrogen (N), Oxygen (O) and Sulfur (S) elements in the structure. Table 1 shows the ultimate analysis of palm biomass with the proximate analysis and lignocellulosic content.

**Table 1:** Lignocellulosic content, ultimate and proximate analysis of palm biomass.

		EFB	PPF	PKS	POME	Ref
Ultimate Analysis (%)	Carbon	45.00–48.79	46.4–51.52	48.06–57.91	50.01	[[8], [9], [10], [11], [12], [13]]
	Hydrogen	5.20–7.86	5.45–9.28	4.77–12.6	15.78	
	Nitrogen	0.25–1.82	0.39–1.89	0.04–1.89	1.99	
	Sulfur	0.36–1.06	0.23	0.05–0.2	–	
	Oxygen	40.18–48.18	40.91–50.21	34.10–49.99	46.90	
Proximate Analysis (%)	Moisture	6.36–8.78	–	5.40–11.00	–	[9,10]
	Volatile matter	71.2–79.65	73.03	67.2–73.77	–	
	Ash	3.00–7.54	10.83	2.1–11.08	–	
	Fixed carbon	8.60–18.30	16.13	15.15–19.7	–	
Lignocellulosic content (%)	Cellulose	38.80–65.00	21.40–49.70	20.8–27.27	–	[8,9]
	Hemicellulose	17.10–33.90	17.10–33.50	21.6–22.7	–	
	Lignin	13.20–25.60	13.20–31.70	44.00–50.70	–	

The energy content of biomass can be estimated from the ultimate analysis. The ratio of the C:H and C:O bonds determine how much energy is given on complete combustion. When the proportion of oxygen and hydrogen is high as compare to that of carbon, the biomass is known to have a lower calorific value (CV) [8]. This is because lower energy is needed to break C:H and C:O bonds as compare to C:C bond. Palm oil biomass have high oxygen content, hence requires less external air for its combustion, a property suitable for economically viable and sustainable fuel source.

Proximate analysis of palm biomass presented them as good sources of bioenergy that can be converted to biofuel using different technologies. The volatile matter and carbon content of palm biomass ranges from 66 to 83 % and 3–20 % respectively, indicating high CV.

Palm biomass is organic and made up of lignocellulose, which is a combination of cellulose, hemicellulose and lignin at different proportions. Hence, can undergo many thermochemical and biological transformations to yield various products [8,9]. The high percentage of cellulose in the biomass makes it a suitable material for the production of composite, pulp and paper.

## 3 Recent researches in the use of palm biomass as precursor for value-adding products

In recent years, many researches have been carried out especially in Malaysia on the waste management of palm biomass owing to the fact that the country is the second largest producer of crude palm oil in the world [14]. The current capacity of Malaysia palm industry is 115.86 million tons of FFB producing huge quantities of waste from the process (statista, 2022). Cameroon is the 7th in terms of palm oil production in Africa, producing about 450,000 tons of crude palm oil annually which is relatively small as compare to Malaysia [[15], [16], [17]]. Although the waste production from the palm sector is comparatively small, however, the subject of waste management should be treated equally by all. Palm waste have been treated either chemically (pulping), thermochemically (pyrolysis), biochemically (aerobic and anaerobic digestions) or biologically (composting and fungiculture) to yield different products, and each method is unique in terms of the process itself, amount of biomass used, carbon footprint, the end-product and usage. This part of the paper will review the available researches on these methods, and the end-products derived from the use of palm biomass as precursor by the Malaysian palm sector/researchers as compare to the Cameroonian palm sectors.

### 3.1 Compost from palm biomass

The direct usage of palm biomass as manure or mulch has lots of advert effect on the ecosystem, such as the presence of contaminants, breeding ground for pest and pathogens, unpleasant smell e. c.t. A better way to mulching with all of its benefits and none of the side effects is composting, it provides a controlled and better path of converting biomass into better quality products that can be used safely [18].

Many Malaysian researchers have documented the conversion of palm biomass into bio-fertilizer either as a better option to mulching or an alternative to chemical fertilizer, since they are environmentally and economically friendly. The feedstock for these processes usually consist of a blend of EFB and POME at different concentrations, and some additive which may include some other type of biomass, fungal, bacterial or even chemical fertilizer. Examples of Malaysian researchers working on this conversion includes Hau et al., 2020 who carried out experiments to produce high quality bio-fertilizer using seven batches of compost derived from EFB, POME, fishmeal, bonemeal, bunch ash and saw dust at different concentrations. The composts achieved maturity on the 40th day, the batches which contained EFB and POME in their mixtures experienced increased in Nitrogen, Phosphorus and Potassium from +18 to 62 %, +125–906 % and +262–294 % respectively. Indicating that the blending of EFB and POME with other organic waste can improve physical properties and C/N ratio of bio-fertilizer [19]. Other researchers like Krishnan et al., 2016 [20] and Lim et al., 2014 [21] had also carried experiments in the composting of palm biomass as a bit to reduce GHG emission to up to 76 % as compare to the methane release during open dumping. Lim et al., 2014 used vermicomposting technique to investigate the suitability of EFB as feedstock to produce bio-fertilizer over a 12 weeks period. The EFB was mixed with cow dung at 5 different ratios and all showed increase in the total contents of Calcium (39.38–373.17 %), Phosphorus (15.15–390.54 %), Potassium (45.55–153.66 %) and Magnesium (55.86–370.93 %) [21].

However, limited researches have either been carried out or published on the same subject of converting palm waste to bio-fertilizers in Cameroon. Yinda et al., 2012 evaluated the composting of EFB and POME biomass from the Idenau oil mill in the enhancement of crop growth [22]. They were able to show that the palm industries in Cameroon could be saving up to 2.5 millionXAF/ha/year if these compost where used instead of urea, generating revenue while solving the waste disposal problems. Despite of this, such knowledge have not been utilized to economically manage the palm waste in Cameroon.

Generally, the composting of palm biomass have following advantages over open dumping: (i) The compost is mainly odorless, reduction in the bulk size and pollution of the EFB waste. Hence, it is easier to manage the waste; (ii) significantly reduces emissions in the form of methane (CH_4_) and carbon dioxide (CO_2_) which are GHGs and contribute to the depletion of the ozone layer; (iii) it increases soil quality and nutrient uptakes (N, P, K, Mg, Ca, Fe, Zn and Cu) by plants. Hence, can be a good alternative to chemical fertilizers and saving money.

The main disadvantage of composting is the time factor which could be as long as 180 days for the compost to mature, and with the plantation scale waste; it is impractical to convert that much to compost. So, there is a need for a faster and more feasible ways of handling these wastes.

### 3.2 Palm biomass as substrate for mushroom cultivation (fungiculture)

Mushrooms are plant-like reproductive structure produced by some fungi. Depending on the type, they can be highly nutritional, flavoured, medicinal and tasty making them useful in human consumptions [23]. Apart from utilizing as food by humans, mushrooms can also be used as animal feed, in the production of beverages, pharmaceutical, nutraceutical and filtration technologies. They grow in the wild and thrive under damp-dark conditions, but have recently been cultivated in a process known as fungiculture. Only 35 out of the 200 identified edible mushrooms are being cultivated commercially, of which Malaysian researchers have exploited this opportunity using palm waste as substrate.

Examples include Yasid et al., 2019 used *G. lucidum* to reduce the cell wall constituents (cellulose, hemicellulose and lignin) of shredded EFB over incubation period of 4, 8 and 12 weeks. After 12 weeks of incubation, EFB fiber composition was reduced from 27.77 to 22.08 % for cellulose, 19.30 to 14.15 % for hemicellulose, while 12.69 to 7.50 % for lignin content. The solution generated serve as a way to convert palm biomass into feed for ruminants [24]. Zakil et al., 2019 another Malaysian researchers investigated the use of EFB, PFF and sugarcane bagasse (SGB) as inexpensive alternative substrate for the cultivation of *P. ostreatus* compared to rubber sawdust (RS). Nine different substrates where prepared from the different combination of the four biomasses. Optimum yield was obtained when the substrate of 25%PPF +25%SGB +50%RS was used. First harvest was done on 35th day, and the yield and biological efficiency (BE) were 318.88 g/kg and 79.72 %, respectively. Thus, proving combination of EFB, PPF and SGB could serve as alternative substrate for the cultivation of *P. ostreatus* when incorporated with RS [25].

Despite the potential of cultivation many tropical edible mushroom in Cameroon, there is little or no information on the use of palm biomass as a substrate for the cultivation of any mushroom type. Chiejina et al., 2015, researchers from neighbouring Nigeria with similar environmental and climatic conditions had proved that it is possible to cultivate edible singer mushroom (*Lentinus squarrosulus*) using PPF and EFB in combination with sawdust as substrate. Maximum yield was obtained when palm fiber was used in the process proving the feasibility of using palm biomass in *L. squarrosulus* cultivation [26].

The above researches proved that palm fiber can serve as alternative and less expensive substrate for the cultivation of different types of mushroom. Therefore, fungiculture is good alternative to the decomposition of palm biomass since they can: (i) quickly decompose organic matter to soil (the most sequestrate form of carbon); (ii) absorb the entire nutrient from the decaying palm biomass, converting it to a consumable form and hence preventing them from escaping to the atmosphere in the form of their oxides; (iii) they are also highly nutritional making them a value adding product. Cameroon palm sector and the communities around the palm plantations could benefit from this knowledge. Adequate researches, sensitizations and trainings should be done in this sector to prepare the local populations on seizing these opportunities.

The main problem with mushroom is their biggest advantage; it lays on the mode of feeding. Mushroom are saprotrophs meaning they can absorb even pollutants and heavy metals like lead, zinc, copper, iron, arsenic, cadmium, mercury and manganese which when consume in large quantities, it poses health risk [[27], [28], [29], [30]]. Even at this, the fact that they can biologically accumulate these heavy metals makes them good bio-indicators and bio-accumulators, and hence eco-cleaners [31].

### 3.3 Palm biomass as raw material for pulp and paper

With the rising environmentally issues poses by the dumping of non-degradable waste like packaging plastics, bags and others; there is a need to promote biodegradable packaging and bags especially from agricultural waste. Pulps being made from the mechanical or chemical stripping of cellulose from lignocellulose biomass are also basic component in the production of paper printout, packaging bags, wraps, napkins and tissues among many others which are renewable, reusable and recyclable.

Usually, EFB and PPF are sometimes mixed with other types of plant fibers or recycled paper in the production process. Examples of Malaysian researchers working on this subject include Hassan et al., 2020, they studied the effect of soda-anthraquinone (soda-AQ) pulping of empty fruit bunches. Optimal result was obtained when 27.3 % NaOH was used at 160 °C for 1 h; the paper produced had 26.8 Nm/g tensile index, 7.95 mN m^2^/g tearing index, 5.32 kPa m^2^/g bursting index, 1.70 log_10_ folding endurance, 46.2 N zero-span tensile strength, 51.8 % brightness, and 95.8 % opacity [32]. Ismail et al., 2020 had also worked on the subject of making paper from palm biomass. Mechanical beating was used incorporation with microcrystalline cellulose (MCC) on EFB; this led to a decrease in porosity and hence increased opacity. The paper made with 6 % of MCC showed the best mechanical properties; and that with beating revolution of 500 showed excellent properties for tear, burst, and folding endurance. Hence, proving that paper derived from the EFB has good potential to be used in the manufacturing of high quality writing and printing paper [33]. More Malaysian researchers working on the subject of reducing the bulk of palm biomass through the process of pulping and paper making includes Liu et al., 2021 [34], Yiin et al., 2019 [35] and Nawawi et al., 2021 [36] hence, proving it feasibilities.

In the case of Cameroon, there is no available information on the use of palm biomass for the process of pulp or paper making, despite the potential. However, researchers from Nigeria Kema et al., 2022 had recently investigated the potential of using EFB for paper making [37]. They were able to obtain a sheeted paper with 80 % abrasion resistance, 180 g/m grammage, with tear strength and tensile strengths of 34.578 Nm^2^/kg and 0.638 kg/cm, respectively. Similar knowledge should be exploited in Cameroon as a means to increase the sustainability of the palm sector and also a means of providing a sustainable source of paper other than deforestation for the same purpose.

Utilizing palm biomass for pulp making will not only replace the process of deforestation for the same purpose but will reduce the bulk of palm waste that are being abandoned in the field to slowly decompose. Hence, reducing GHG emission and generating revenue from the process. Also, pulp are degradable, renewable and recyclable making them a better alternative to plastic packaging that have polluted are land and sea, and creating a hazardous environment to the entire ecosystem.

### 3.4 Palm biomass for char, activated carbon and nano carbon production

Another way of converting palm biomass into a value added product is through the process of ‘Carbonization’ also known as pyrolysis which is the thermochemical decomposition of organic material at high temperatures in the absence of oxygen [38]. Thermochemical decompositions are faster as compare to other types and the product obtained (char) can be used in different application not limited to fuel. Char can also be applied in soil amendment, water treatment, pharmaceutical, air cleaner, silage agent, bio-fertilizer, production of nano-materials, and electrochemical electrodes among others.

Recent research on the use of palm biomass as source of bio-char production include Abdullah et al., 2015, they were able to show that carbonized palm waste have potential of replacing coal as fuel. The palm biomasses used in their experiment were PKS, PPF and EFB, subjected under experimental conditions of temperature 300–500 °C, 2 h retention time and heating rate of 10 °C/min. The bio-char obtained showed a great potential to be used as replacement of coal, as well as soil improvement and activated carbon applications [39]. Nalaya et al., 2020 are another Malaysian researchers working on the adsorption of pentachlorophenol (PCP) using carbonized EFB. EFB carbonized at 350 °C was able to adsorb this harmful PCP, with the maximum adsorption capacity equals to 6.035 mg/g [40].

Nano carbons have also been documented to be produced from palm biomass. Mamun et al., 2018 produced carbon nano-tubes from PKS; the furnace temperature was range from 550 to 850 °C using iron as catalyst. The nano carbon obtained had particle size between 25 and 35 nm [41]. Another type of nano carbon, graphene had also been produced from palm biomass. Graphene have high conductive properties, making them very useful in the manufacturing of nano-electronics and embedded systems [[42], [43], [44]].

Thermochemical decomposition has a major advantage in terms of time; unlike biological and chemical decomposition that might take months to complete, (i) pyrolysis takes shorter time (as short as minutes) and (ii) the product obtained (char) can be used in different application not limited to fuel.

There is no available publication on the same subject in Cameroon, despite its long range of applications and its ability to quickly manage this industrial size waste.

The main disadvantages of this conversion method are the temperature dependency and the occasional usage of chemical. The process uses high energy profile of up to 1000 °C in its conversion, and chemicals which could result to chemical pollution if not handled with care.

### 3.5 Palm biomass as an alternative source of energy

Fossil fuel emit GHGs that have adverse effect to man and the environment, it will also be completely depleted someday; hence, there is a need to promote the use of biofuel which are not only clean but also replenishable. Apart of bio-char, palm biomass can be converted to other types of biofuel like bioethanol, biodiesel, bio-hydrogen and others using many different methods which include fermentation, enzymatic hydrolysis, anaerobic digestion, catalytic cracking and *trans*-esterification, plus the thermochemical processes mention above [45].

Recent researches on the use of palm biomass for the production of bioenergy includes Selvarajoo et al., 2021, they produced bio-pellet by mixing different ratio EFB and durian fiber. The sample was first carbonized at 325 °C, for 2 h, at heating rate of 5 °C/min to obtained bio-char. Then, the bio-char were pelletized using 1 % corn starch as adhesive. The mixture of 90 % EFB and 10 % durian gave the maximum yield of 22.37 MJ/kg. The pellets produced were stable and firm, making them easier to store, handle and transport as compared to the bio-char. Loh et al., 2018, are another Malaysian researchers working on the fermentation of EFB to produce bioethanol. Based on their experiments, maximum bioethanol of 0.66 g/g glucose was obtained when the optimized condition of pH 4, 30 °C, 150 rpm and 72 h was used for the fermentation [46]. Many other forms of bioenergy have been produced from palm biomass [47].

In Cameroon, PKS and PPF are almost completely used up in the mills, with small quantities of these wastes being given out to the communities and workers of the oil mills to be used as biomass. The local communities closed to the mills always have access to some kind of specialized stoves that are designed for the utilization of this biomass to produce cooking energy. For those who use ‘three-stone fire’, PKS and PPF are being used as fire starter. Researches on the conversion of EFB to any form of biofuel in Cameroon are limited. More researches need to be done to completely utilized palm waste especially EFB to other form of bioenergy, hence cleaner environment.

### 3.6 Palm biomass for construction material production

With the ever-increasing real estate and construction projects, and the increase in cost and pollution from the processes; there is a need to go green in construction. One way of reducing the cost of construction, pollution and increase sustainability in this sector is to use plant fibers (biomass) as part of construction material [48]. These fibers have been used in the manufacturing of furniture, flooring, wall-partitioning, cementing, concreting and false ceilings among others [49]. Some examples of plant fibers that have been used for these purposes includes bamboo, hemp, jute, sugarcane, sisal, date palm and coconut shell fibers among others using different treatment processes [50,51]. Palm biomass have the physical and mechanical characteristics that are needed for it to be utilized as precursor for the development of construction materials [52].

Kaliwon et al., 2010 constructed reinforced roofing slates from empty palm bunches. The flexural strength of the roofing slate increased to 4.66 N/mm^2^, a value greater than the ASTM standard of 4 N/mm^2^ when 0.5 % of the EFB fiber was used in the mixture, while the density decreased; hence, demonstrating the potential of EFB fiber to be a good material for the construction of roofing slates [53]. Palm oil fuel ashes (POFA), residue left after complete combustion of PKS and PPF in the mills have also been documented in the development of composites because they are pozzolanic in nature as shown by Raut et al., 2016 [48,49]. They demonstrated that POFA can be used to partially substitute cement to up to 10 % replacement.

EFB and PPF have served as alternative material to production of fiberboards, particleboards, and concrete in Malaysia. Despite these potential, there is no available research on the use of palm biomass to produce any type of construction materials in Cameroon. Construction materials made from palm biomass are limited to roof, fencing and broom-making which all utilized palm fronds and it is mostly in rural areas [54].

Composite derived from palm biomass, like all other plant fiber have the advantages of: (i) reduction in terms of cost of materials (Green building) and environmental hazard; (ii) increase in the flexural and tensile strength of the concrete; (iii) and increase in the thermal insulation capacity and hence saves energy that would have been used for heating.

The main disadvantages of these composite are: (i) increase in porosity of the material, leading to increase in water absorption and decrease to compressive strength. In a long run, the composite might collapse; (ii) lengthy curing period of up to 180 days [55]; (iii) small quantities of the fiber are being utilized in the process, it may be as low as 0.5 % as compared to the entire weight of the concrete.

### 3.7 POME as precursor for value-adding products

The recent trends in the conversion of POME into value adding products mainly focus on digestions (anaerobic and aerobic) to the production of biofuel (bio-methane, bio-hydrogen, bioethanol, biodiesel etc). Current research on this include; Said et al., 2020 [56] who used a consortium of bacteria to investigate the quantity of methane produce from the anaerobic decomposition of POME. Based on this, the highest production of methane achieved was 41.05 % when *Bacillus toyonensis* (strain BCT-7112) and *Stenotrophomonas rhizophila* (strain e-p10) were used in an 18-days anaerobic fermentation process. The highest reduction of chemical oxygen demand (COD), biological oxygen demand (BOD) and total suspended solid (TSS) recorded were 86, 94 and 80 %, respectively; and the bacterial strived at the pH range of 7.8–8.3.

Ghosh et al., 2020., also investigated the accumulation of polyhydroxyalkanoates (PHA) biopolymers from the aerobic digestion of POME in the presence of *Micrococcus aloeverae (*strain SG002) [57]. PHA/mg cell dry weight yielded 0.71 ± 0.04 mg when a daily change of the organic loading was 0.6–1.8 kg COD/m^3^. PHA accumulated in the feast phase was in the range of 40–70 %, this was utilized in the famine phase. PHA yield per unit COD removal ranged between 0.38 and 0.89 mg PHA/mg COD. The studies showed a suitable method of treatment of POME; and PHA has also been documented in the production of bio-packaging, biofuel, biomedical implants, tissue engineering and others [[58], [59], [60]].

POMEs are another waste disposed by the oil mills in Cameroon usually in an unsustainable manner. The POME and wastewater from the plants are being disposed at temperatures greater than 60 °C to water bodies like the sea in the Idenau local area or on land like the case of Mondoni oil mill [61]. These have an adverse effect to the environment and the ecosystem in the disposal area causing land pollution and destruction of marine organism [22]. For those agro-industrial palm producers who claimed to have built ponds for the treatment of POME, there is no methane capture facility, example include SOCAPALM (socapalm, 2020). Methane capture and treatment facilities should have been built to capture and treat the gas to be used as cooking gas. Cameroonian researchers Toh et al., 2014, worked on the anaerobic digestion of EFB and POME to biogas. The EFB and POME were mixed at different concentrations in the presence of boiler ash, at a digestion time of 25 days under mesophilic atmosphere; the methane content obtained was greater than 50 % [62]. Further researches could have been carried out for the optimization and purification of this methane to provide cooking gas.

The women in the local communities have evolved in the process of converting these POMEs waste into a source of income. Waste oil catchment pondsare usually constructed a few meters from the mills and the waste oil is being trapped for commercial purposes. ‘Gutter-oil’ as it is called is further process by heating to be sold to some soap and cosmetic companies as raw material for their production processes.

## 4 Sustainability and feasibility of the various conversion processes

As a guide for future researchers working on the conversion of palm biomass into useful product, this section summarizes the various conversion processes in terms of sustainability and feasibility to come out with a grading system serving as advice. The sustainability of the process will be quantified as low, medium and high depending on.

• Energy consumption profile (can it run green?)
• Waste generation from the process (eco-friendly)
• Carbon footprint reduction

The feasibility of the process will be measured in terms of.

• How long it takes to complete the conversion process
• How much quantity of biomass is used per conversion process

Priority scheme of the processes depend on the sustainability and feasibility of each, together with possible application to the end product. End-product like biofuel has high priority because of the ready market for energy and its importance in the development of sustainable environment. Bio-char from pyrolysis takes hours to complete, and the end product has a wild range of application in many sectors, making the thermochemical process of high priority in conversion of biomass into value-adding product. Compost on the other hand takes up to 12 weeks to mature and the end-product is limited to fertilizer. So, the priority of this conversion should be low. Same can be said to composite which uses less than 0.5 % of the fiber per process and has a long curing time of up to 180 days. Table 2 summarizes the various palm waste management in terms of sustainability, feasibility and priority of the processes.

**Table 2:** Sustainability and Feasibility studies of the various palm management processes.

Process	Sustainability	Feasibility	Comments/Priority	Ref
Anaerobic digestion	Green process Reduction in carbon print and zero toxic waste High Sustainability.	Takes a period of 10–30 days Takes as much quantity as the size of the digester Highly feasibility	Ready market for end product (biofuel) High Priority	[22,62,63]
Aerobic digestion	High cost of aeration process Reduction in carbon print and zero waste Medium Sustainability	Takes shorter time as compared to anaerobic process Takes as much quantity as the size of the digester Highly feasibility	End product (Ammonia) has limited application Medium Priority	[61,64]
Composite	Green building Reduction in carbon print and zero waste High Sustainability.	Takes a long time mature (up to 180 days) Use small percent of the biomass (0.5 % or less per process) Low feasibility	Long curing time and uses small percentage of fiber Low Priority	[[48], [49], [50], [51], [52],^54^,^65^]
Compost	Green process Reduction in carbon print and zero toxic waste High Sustainability.	Takes a long time to mature (up to 12 weeks) Can be reproduced in a large scale Medium feasibility	Limited use of the end- product. Long time to mature Medium Priority	[[19], [20], [21], [22],^66^]
Pyrolysis	Use high temperature profile Reduction in carbon print and zero waste Medium Sustainability	Takes short time (as short as minutes) complete Use high percentage of biomass Highly feasibility	End-product (bio-char) has wide range of application. Takes hours to complete High Priority	[40,[67], [68], [69], [70]]
Mushroom cultivation	Green process Reduction in carbon print and zero toxic waste High Sustainability.	Takes about 35 days to mature and harvest. Slow decomposition Use small percentage of biomass low feasibility	End-product has wide range of application Slow decomposition process Medium Priority	[[24], [25], [26],^71^]
Paper and pulp	Green process Reduction in carbon print and deforestation Biodegradable and recyclable High Sustainability.	Takes a day or two mature Can be reproduced in a large scale Medium feasibility	Wide range of application Ready market for the end-product High Priority	[[32], [33], [34], [35], [36], [37],^72^]

The aspect of the actual need of the end-product is dependent on the end-user consumers/government base on their need, resources available and the market. This scaling system is good but on the subject of sustainability, any of these processes can be employ to manage agro-industrial waste regardless of the priority. Feasibility here explained the possibility of the system to be able to transform huge volume of waste. The conversion time and quantity says a lot about the waste managements. On this scale, the most feasible process is pyrolysis (thermochemical). It is not only the fastest but produces biochar/biofuel which can be used for wide range applications.

### 4.1 Recommendation and way forward

When it comes to the subject of climate change, sustainability and development, there are no little steps. Cameroon should take steps pass the local utilization of this biomass to a more sustainable and feasible ways in managing His agro-industrial waste, seeking advice from Table 2 above together with the actual need of the end-product.

The palm oil industries in Cameroon should make public the recent waste management techniques that have been adopted, paying more attention to EFBs and POMEs. The industries should present sustainable waste disposal processes, including the construction of methane capture facilities to produce biofuel for the communities and help the country emerge.

The Roundtable on Sustainable Palm Oil (RSPO) rules and regulations should be followed to the latter to ensure sustainability in the palm sector. Any further investment in this sector should be sustainable in terms of the ecosystem, land acquisition, treatment of workers and health care, addressing all the present issues being faced by the existence agro-industrial palm companies in Cameroon.

New investors in the palm sector in the country should provide a whitepaper with information that tackling all the sensitive issues that have been hunting the sustainability of palm oil industries.

## 5 Conclusion

Palm oil wastes have high potential to not only to be managed sustainably but also to be economically viable. Researchers from Malaysia have proving the feasibility of sustainably handling of palm waste; same cannot be said about Cameroon and other palm producing Nations in Africa. Apart from the small quantities of the biomass used by the locals and the immediate communities close to the mills. There had not been adequate researches on the subject nor massive utilization of this waste; hence, the palm waste management is non-sustainable. Cameroon and other Nations in Africa producing palms should learn from this sustainable palm waste management.

## Data availability statement

Data will be made available on request.

## CRediT authorship contribution statement

**Egbe Terence Awoh:** Conceptualization, Data curation, Formal analysis, Investigation, Methodology, Resources, Software, Visualization, Writing – original draft, Writing – review & editing. **Joseph Kiplagat:** Conceptualization, Formal analysis, Project administration, Supervision, Validation, Visualization, Writing – review & editing. **Stephen K. Kimutai:** Conceptualization, Data curation, Formal analysis, Methodology, Resources, Supervision, Validation, Writing – review & editing. **Achisa C. Mecha:** Conceptualization, Data curation, Formal analysis, Investigation, Methodology, Software, Supervision, Validation, Writing – review & editing.

## Declaration of competing interest

The authors declare that they have no known competing financial interests or personal relationships that could have appeared to influence the work reported in this paper.
